# Clinical Use and Treatment Mechanism of Molecular Hydrogen in the Treatment of Various Kidney Diseases including Diabetic Kidney Disease

**DOI:** 10.3390/biomedicines11102817

**Published:** 2023-10-17

**Authors:** Shin-ichi Hirano, Yusuke Ichikawa, Bunpei Sato, Yoshiyasu Takefuji, Fumitake Satoh

**Affiliations:** 1 Department of Research and Development, MiZ Company Limited, 2-19-15 Ofuna, Kamakura 247-0056, Japan; y_ichikawa@e-miz.co.jp (Y.I.); b_sato@e-miz.co.jp (B.S.); f_satoh@e-miz.co.jp (F.S.); 2 Keio University, 2-15-45 Mita, Minato-ku, Tokyo 108-8345, Japan; takefuji@keio.jp; 3 Faculty of Data Science, Musashino University, 3-3-3 Ariake, Koto-ku, Tokyo 135-8181, Japan

**Keywords:** molecular hydrogen, diabetic kidney disease, mitochondrial dysfunction, oxidative stress, inflammation, reactive oxygen species, medical application

## Abstract

As diabetes rates surge globally, there is a corresponding rise in the number of patients suffering from diabetic kidney disease (DKD), a common complication of diabetes. DKD is a significant contributor to chronic kidney disease, often leading to end-stage renal failure. However, the effectiveness of current medical treatments for DKD leaves much to be desired. Molecular hydrogen (H_2_) is an antioxidant that selectively reduces hydroxyl radicals, a reactive oxygen species with a very potent oxidative capacity. Recent studies have demonstrated that H_2_ not only possesses antioxidant properties but also exhibits anti-inflammatory effects, regulates cell lethality, and modulates signal transduction. Consequently, it is now being utilized in clinical applications. Many factors contribute to the onset and progression of DKD, with mitochondrial dysfunction, oxidative stress, and inflammation being strongly implicated. Recent preclinical and clinical trials reported that substances with antioxidant properties may slow the progression of DKD. Hence, we undertook a comprehensive review of the literature focusing on animal models and human clinical trials where H_2_ demonstrated effectiveness against a variety of renal diseases. The collective evidence from this literature review, along with our previous findings, suggests that H_2_ may have therapeutic benefits for patients with DKD by enhancing mitochondrial function. To substantiate these findings, future large-scale clinical studies are needed.

## 1. Introduction

Kidney diseases include a wide variety of conditions, including glomerulonephritis and pyelonephritis caused by kidney inflammation and infection, as well as nephrosclerosis, diabetic kidney disease (DKD), and polycystic kidney disease caused by hypertension, atherosclerosis, and diabetes mellitus. DKD is one of the complications of diabetes mellitus and is also a major cause of chronic kidney disease (CKD) progressing to end-stage renal disease (ESRD) [1,2].

The International Diabetes Federation estimated that the global prevalence of diabetes mellitus would reach 10.5% (536.6 million individuals) in 2021 and 12.2% (783.2 million individuals) by 2045 [3]. With the rapid increase in diabetes mellitus worldwide, the number of patients with DKD also continues to rise, particularly in developed countries [1,2,3]. DKD is a risk factor not only for progression to ESRD but also death from cardiovascular complications [4]. Approximately 30–40% of patients with diabetes mellitus develop DKD, making it a global public health and health economic issue [1,2,3].

Molecular hydrogen (H_2_) is a flammable, colorless, and odorless gaseous molecule. It functions as an antioxidant that directly reduces hydroxyl radicals (·OH) and peroxynitrite (ONOO−), which are reactive oxygen species (ROS) and reactive nitrogen species (RNS), respectively, with very potent oxidative capacities [5]. In addition, H_2_ exerts a number of indirect effects, including antioxidant, anti-inflammatory, and cell lethality-regulating effects, through the regulation of gene expression, in addition to its effects through nuclear factor erythroid-related factor 2 (Nrf-2) and intracellular signal transduction [6,7,8,9]. The total number of publications on the biological effects of H_2_ currently exceeds 2000. H_2_ not only crosses the blood-brain barrier easily, but also biological membranes to reach mitochondria and protect cells from ROS- and RNS-induced cell damage [10]. Although the target molecules of H_2_ remain unknown, oxidized porphyrins were recently shown to catalyze the reaction of H_2_ with ·OH, thereby reducing oxidative stress [11].

Many factors are involved in the development and progression of DKD, among which mitochondrial dysfunction, oxidative stress, hyperglycemia, and inflammation have been strongly implicated [12,13,14,15]. Recent preclinical and clinical studies reported that new therapeutic agents that reduce oxidative stress may slow the progression of DKD [16,17,18,19,20]. Other studies demonstrated the efficacy of H_2_ in various animal renal disease models [21,22,23,24,25,26,27,28,29,30,31,32,33,34,35,36,37,38,39,40,41,42,43]. Furthermore, H_2_ gas inhalation therapy or therapy with H_2_-enriched dialysis solution was shown to attenuate oxidative stress in dialysis patients [44,45,46,47]. However, to the best of our knowledge, the potential therapeutic effect of H_2_ on DKD has not been reported. Therefore, since it is important to develop new substances with superior efficacy and safety that demonstrate therapeutic potential against DKD, we hypothesized that H_2_ may ameliorate the development and progression of DKD through its effect on improving mitochondrial function. Based on this hypothesis, we aimed to analyze the literature in which H_2_ has demonstrated efficacy in various animal models of renal disease and in human dialysis patients [21,22,23,24,25,26,27,28,29,30,31,32,33,34,35,36,37,38,39,40,41,42,43,44,45,46,47]. Moreover, by integrating this literature analysis with our previous mechanistic studies on the efficacy of H_2_ against chronic inflammatory diseases [48,49], we explored the potential therapeutic benefits of H_2_ for DKD. We present this paper with the aspiration that this review will stimulate consideration for potential clinical research on the application of H_2_ in treating DKD.

## 2. Methodology

**Figure 1:**
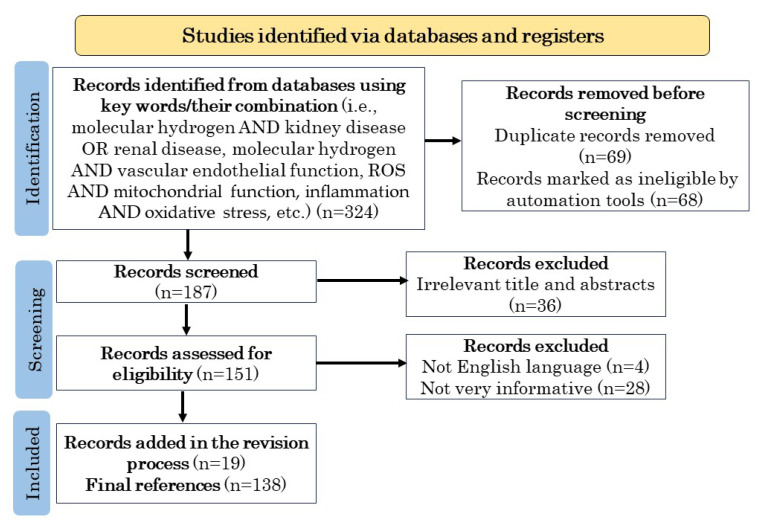
PRISMA flowchart describing the process of published data selection.

To investigate the therapeutic potential of H_2_ for DKD, we conducted a comprehensive literature search focusing on the etiology of DKD and current treatment methods and its efficacy, the relationship between oxidative stress and inflammation, ROS production and elimination, the relationship between ROS and pathogenesis, mitochondrial function, the involvement of ROS in renal disease, the effects of H_2_ on animal models of renal disease and human patients, and the effects of H_2_ on vascular endothelial function. From April 1991 to September 2023, we utilized electronic databases from PubMed and Google Scholar for our research. In our PubMed search, we employed Medical Subject Headings (MeSH) terms. Our search strategy involved the use of Boolean operators AND, OR, and NOT to combine keywords. The PRISMA flowchart in Figure 1 describes the methodology for already published information selection, using the directions given by Page et al. [50,51].

## 3. H_2_ Regulates Oxidative Stress

### 3.1. History and Progress of Research on Medical Applications of H_2_

In 1975, Dole et al. reported that H_2_ gas exerted anti-tumor effects. They showed that the inhalation of an 8 atm mixture of 2.5% oxygen and 97.5% H_2_ significantly regressed squamous cell carcinoma in mice [52]. In 1994, Abraini et al. demonstrated that the inhalation of a mixture of 49% H_2_, 50% helium, and 1% oxygen effectively prevented decompression sickness in divers [53]. In 2001, Gharib et al. showed the anti-inflammatory effects of hyperbaric H_2_ gas in a mouse model of chronic hepatitis induced by schistosomiasis infection [54]. Furthermore, in 2005, Yanagihara et al. found that saturated concentrations of neutral H_2_ water produced by water electrolysis reduced chemical oxidant-induced liver injury in rats [55].

In 2007, Ohsawa et al. reported the utility of H_2_ as a therapeutic antioxidant by selectively reducing ·OH and ONOO−, which have very potent oxidative capacities [5]. Medical applications of H_2_ have since attracted worldwide attention and marked advances have been achieved in this field. In 2023, oxidized porphyrins were shown to catalyze the reaction of H_2_ with ·OH [11]. A mechanism was proposed in which the potent oxidative capacity of ·OH is reduced in the presence of H_2_ and porphyrins, which activates Nrf-2 as a hormesis-like effect and induces the antioxidant enzyme heme oxygenase-1 (HO-1) [56]. However, given that the only data reporting reactions of H_2_ with mammalian tissues or biomolecules are found in the literature on iron porphyrins, it is clear that research on the target molecules of H_2_ is still in its early stages.

### 3.2. ROS Production and Scavenging Systems

**Figure 2:**
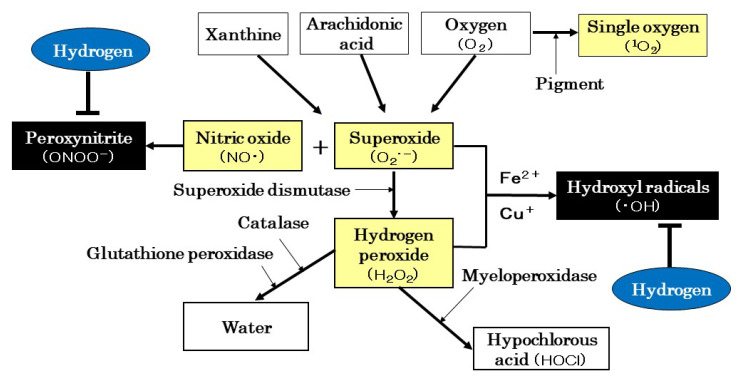
ROS production and scavenging systems. Antioxidant enzymes, such as superoxide dismutase, catalase, and glutathione peroxidase, cannot scavenge ∙OH and ONOO^−^, which are potent oxidants. In contrast, H_2_ selectively scavenges ∙OH and ONOO^−^, converting them to water. ROS: reactive oxygen species; ∙OH: hydroxyl radicals; ONOO^−^: peroxynitrite.

Oxygen is essential for the production of energy by breathing organisms and is used by the mitochondria of cells to generate adenosine triphosphate (ATP). However, 1–2% of consumed oxygen becomes ROS, which has potent oxidative properties [8,10,57,58]. ROS in humans is mainly superoxide, hydrogen peroxide, ·OH, and singlet oxygen [8,10,57,58]. Superoxide is formed when electrons leaking from the mitochondrial respiratory chain combine with oxygen. Superoxide is also produced by xanthine oxidase, which uses oxygen and xanthine as substrates, or the arachidonic acid cascade in vascular endothelial cells. Superoxide is a relatively potent ROS but is degraded to hydrogen peroxide by superoxide dismutase (SOD) [8,10,57,58]. Hydrogen peroxide is further decomposed into water and oxygen by catalase (CAT) and glutathione peroxidase (GPX) [8,10,57,58]. On the other hand, singlet oxygen is generated when oxygen reacts with pigments in the body that act as sensitizers when the organism is exposed to ultraviolet radiation (Figure 2).

The human body is equipped with antioxidant enzymes, such as SOD, CAT, and GPX, as described above, as a defense mechanism to suppress the formation of ROS. However, the function of antioxidant enzymes and the body’s defense capabilities against ROS decline with age [10,58]. Furthermore, when ROS are produced in large amounts due to mental and physical stress, excessive exercise, smoking, and exposure to ultraviolet light and irradiation, the balance between ROS production and scavenging systems is disrupted, and ROS that exceed the protective capacity of antioxidant enzymes appear [10,58]. The disruption of the balance between oxidation and anti-oxidation results in superoxide and hydrogen peroxide catalyzed by iron and copper ions producing ·OH, which has a very potent oxidative capacity [59]. ·OH is also generated in other biological reactions and when water, a biological substance, is exposed to irradiation. ·OH is present in the body for only one millionth of a second; however, during that time, it exhibits an oxidizing power 100-fold stronger than that of superoxide [60]. On the other hand, nitric oxide (NO·) reacts with superoxide to produce ONOO−, which is extremely oxidizing [8,57]. When ·OH and ONOO− are produced, they react with nucleic acids, lipids, and proteins in biological membranes and tissues, causing oxidative damage and even oxidizing DNA. Antioxidant enzymes cannot scavenge ·OH and ONOO−, while H_2_ selectively reduces ·OH and ONOO−, converting them to water (Figure 2) [8,10,57,58].

### 3.3. “Beneficial” and “Detrimental” Effects of ROS

ROS have both “detrimental” and “beneficial” effects on organisms. Superoxide and hydrogen peroxide are cytotoxic at high concentrations, but function as second messengers in signal transduction mechanisms and as regulators of immune cell metabolism, activation, cell proliferation, cell differentiation, and apoptosis at low concentrations [10,58]. Hydrogen peroxide at high concentrations is converted by antioxidant enzymes to hypochlorous acid, which protects the body from bacterial attack. NO· is important for intracellular signal transduction and vasodilation and is used clinically as a medical gas. On the other hand, a difference between eustress and distress has been reported. Large amounts of ROS cause oxidative damage, whereas small amounts of ROS activate Nrf-2 and induce HO-1, exerting antioxidant effects [9,61]. Small amounts of ROS also induce the expression of the tumor suppressor gene, p53, which plays an important role in the prevention of cancer [62]. Furthermore, small amounts of ROS are crucial for maintaining homeostasis in organisms [63].

Oxidative stress caused by ROS causes mutations in normal cells and promotes their transformation into cancer cells. Therefore, a method to remove ROS and reduce oxidative stress by antioxidants was considered promising for cancer prevention and treatment. A large clinical trial on vitamin E supplementation was conducted [64,65]. However, contrary to expectations, the incidence of prostate cancer was significantly higher in patients treated with vitamin E [64,65]. Studies using mouse models of cancer also reported the cancer-promoting effects of N-acetylcysteine and vitamin E supplementation [66]. The mechanism by which these antioxidants promote cancer growth was elucidated by DeNicola et al. and Schafer et al. [67,68]. Therefore, the effects of antioxidants on cancer are two-sided, either inhibiting or promoting carcinogenesis, depending on the conditions. H_2_ has not yet been shown to exert carcinogenic effects, whereas its carcinogenic inhibitory effects have been demonstrated [69].

**Figure 3:**
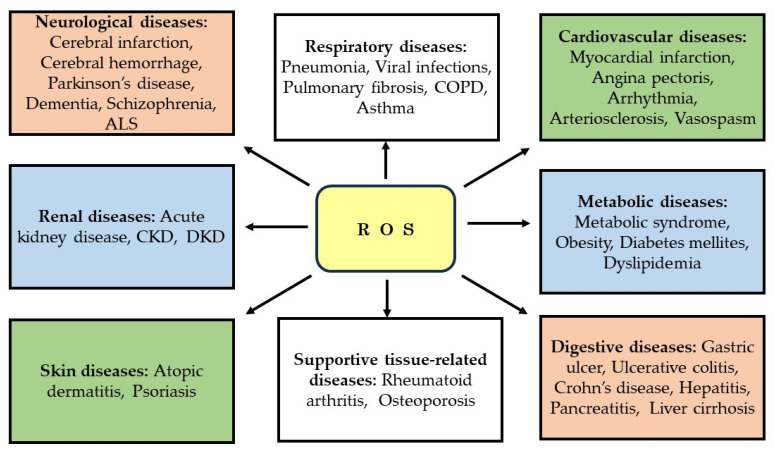
ROS involvement in various diseases. ALS: amyotrophic lateral sclerosis; COPD: chronic obstructive pulmonary disease; CKD: chronic kidney disease; DKD: diabetic kidney disease; ROS: reactive oxygen species.

ROS play a significant role in the onset and progression of not only DKD but also a vast array of other diseases. These include neurological diseases such as cerebral infarction, cerebral hemorrhage, Parkinson’s disease, dementia, schizophrenia, and amyotrophic lateral sclerosis, as well as cardiovascular diseases like myocardial infarction, angina pectoris, arrhythmia, arteriosclerosis, and vasospasm [70,71]. Involvement of ROS is also observed in respiratory diseases such as pneumonia, viral infections, pulmonary fibrosis, chronic obstructive pulmonary disease, and asthma, renal diseases such as acute kidney disease, CKD, and DKD, and digestive diseases such as gastric ulcer, ulcerative colitis, Crohn’s disease, hepatitis, pancreatitis, and liver cirrhosis [70]. Furthermore, ROS involvement is also observed in metabolic diseases such as metabolic syndrome, obesity, diabetes mellites, and dyslipidemia, skin diseases such as atopic dermatitis and psoriasis, and supportive tissue-related diseases such as rheumatoid arthritis and osteoporosis [70,71]. Therefore, it would not be an overstatement to say that ROS are implicated in diseases affecting most organs and tissues throughout the human body (Figure 3).

## 4. Mitochondrial Involvement in Renal Disease

### 4.1. Mitochondrial Structure and Function

Mitochondria are intracellular organelles that produce more than 90% of intracellular energy and generate ATP by oxidative phosphorylation (OXPHOS) under aerobic conditions [8,10,54,58]. Mitochondria comprise an outer membrane, inner membrane, intermembrane lumen between the outer and inner membranes, a matrix surrounded by an inner membrane, and a crista lumen surrounded by a recessed crista membrane [72]. Mitochondria have their own genome, mitochondrial DNA (mtDNA), which is distinct from the nuclear genome (nDNA) [72]. This is attributed to the intracellular parasitism of the aerobic bacterium, *Proteobacteria*, into archaea approximately 2 billion years ago, which transformed them into mitochondria [73]. Therefore, the structure of mtDNA is more similar to the bacterial genome structure than to the eukaryotic nuclear genome. Mitochondrial respiratory chain complexes, complexes I-V, assemble in inner membrane cristae for efficient ATP production [72,74,75]. Many mitochondrial ROS (mtROS) are generated during this ATP production process, mainly from complexes I and III, which are normally removed by the antioxidant system [72,74,75]. Therefore, mitochondria maintain efficient energy production by the electron transfer system of the inner membrane respiratory chain complex and OXPHOS by ATP synthase.

### 4.2. Role of ROS in Renal Disease

Mitochondrial disease is caused by abnormalities in various genes involved in mitochondrial function and structural maintenance, including ATP synthesis, the transport of amino acids, lipids, and proteins, and the removal of oxidative stress within mitochondria [74,75]. These genetic abnormalities may result from both mtDNA aberrations and genetic mutations in nDNA [76]. When mitochondrial function is impaired because of these genetic abnormalities, organs with high energy demands become dysfunctional and exhibit a wide range of symptoms [74,77]. Mitochondrial diseases are called mitochondrial encephalomyopathies or mitochondrial myopathies, depending on the location of the disorder [78].

Abnormal mitochondrial function is induced by various factors other than genetic abnormalities that decrease ATP production and increase mtROS, and cell lethality is caused by apoptotic signals, such as cytochrome c released from within damaged mitochondria [75,77]. This type of mitochondrial dysfunction has been observed in many metabolic and neurodegenerative diseases [79]. Among them, the kidney, which has the highest volume per tissue weight and oxygen consumption among organs after the heart and brain, respectively, relies upon mitochondrial ATP production for active mass transport energy metabolism in the tubular epithelium [75,80]. Furthermore, since podocytes, the glomerular vascular endothelium, and mesangial cells, which perform glomerular filtration functions, are active in mitochondrial metabolism, the kidney is subject to the failure of energy metabolism due to ischemia, hypoxia, and mitochondrial dysfunction from toxic substances [81,82,83,84,85].

With increases in the number of patients with diabetes mellitus, DKD has become the leading disease among ESRD [1]. Diabetes mellitus is considered to be the primary cause of the recent increase in the number of patients on dialysis [1,2,3,4]. It decreases the effects of insulin in cells, resulting in chronic hyperglycemia and dyslipidemia [86,87]. Therefore, abnormalities in signaling mechanisms, such as nutrient-sensitive AMP-activated protein kinase (AMPK) and mechanistic target of rapamycin complex 1, and mitochondrial dysfunction are observed [86,87]. In addition, oxidative stress is increased in diabetes mellitus due to the intracellular influx of excess glucose, which promotes the mitochondrial production of excess mtROS, activates the polyol metabolic pathway, activates protein kinase C, and leads to the accumulation of advanced glycation end products [88,89,90]. Oxidative stress also promotes the development of DKD.

However, physiological levels of ROS are essential for the regulation of intracellular signaling mechanisms and cellular homeostasis [10,58]. Mitochondrial membrane potential and respiratory regulation rates are reduced in diabetes mellitus and, conversely, ROS production is decreased, which inhibits OXPHOS and ATP production [86,91]. Continued reductions in OXPHOS and ATP production in mitochondria increase oxidative stress outside mitochondria due to the uncoupling of nicotinamide adenine dinucleotide phosphate (NADPH) oxidase and endothelial-type NO· synthase, releasing cytokines involved in inflammation and fibrosis and inducing cellular damage [92]. ROS derived from the cytoplasm are considered to play a more important role than mtROS in this case. The administration of AMPK activators to animal models of diabetes mellitus was previously shown to improve mitochondrial function and induce the production of mtROS, thereby decreasing albuminuria and attenuating DKD [86,92,93]. As part of the mitochondrion-targeted DKD therapeutic strategy, the utility of a treatment that improves mitochondrial function and induces physiological levels of ROS production has been proposed [92,93].

### 4.3. Development Status of Diabetic Kidney Disease Therapeutics

Bardoxolone methyl (BM) has attracted the most attention as a potential treatment for DKD. It is a novel synthetic triterpenoid with an oleic acid skeleton [16]. BM was initially developed as an anticancer agent [94]. Phase I clinical trials on cancer patients showed an increase in the estimated glomerular filtration rate (eGFR); therefore, BM was converted into a DKD treatment [94]. The main mechanism by which BM improves renal function is the activation of the Kelch-like ECH-associated protein 1 (Keap1)/Nrf-2 pathway [16]. After a phase II open-label and double-blind placebo-controlled trial, a phase III double-blind placebo-controlled trial (AYAME study) in Japan was conducted to prove the efficacy and safety of BM [95]. However, although this clinical trial showed an improvement in eGFR, it failed to demonstrate that BM inhibited the development of ESRD, and, thus, further research on BM was discontinued [96]. Other substances that have been reported to exhibit similar mechanisms to BM include curcumin, isothiocyanate, cinnamic aldehyde, resveratrol, and α-lipoic acid [97,98,99,100,101]. These substances are in preclinical or early clinical trials and are expected to be developed as activators of the Keap1/Nrf-2 pathway in the future. In addition, sodium-glucose contransporter-2 (SGLT2) inhibitors exert their effects on oxidative stress and inflammation, while glucagon-like peptide 1 (GLP-1) receptor agonists act on the AMPK-mammalian target of rapamycin (mTOR)-autophagy-ROS-signaling axis, and both were shown to be effective in animal models of DKD [17,18]. These SGLT2 inhibitors and GLP-1 receptor agonists are expected to be useful as therapeutic agents targeting oxidative stress.

On the other hand, DKD therapeutics that target an improvement in mitochondrial function have been developed. One highly mitochondrion-directed antioxidant is coenzyme-Q (MitoQ), which was found to be effective in animal models of ischemia-reperfusion (I/R) injury and DKD [19,102]. However, MitoQ has a number of limitations, such as becoming an oxidant itself, particularly at high doses, which reduces its efficacy [87,90,103]. Elamipretide (SS-31), which acts on cardiolipins in the mitochondrial inner membrane, was also effective in I/R injury and DKD models, and various clinical trials on SS-31 in DKD patients are ongoing [104]. Mitochonic acid 5 (MA-5), a novel synthetic indole compound characterized by its ability to increase intracellular ATP and decrease mtROS, has also attracted interest [105]. MA-5 was effective against I/R nephropathy and cisplatin-induced nephropathy [20]. It also improved cardiac and renal mitochondrial respiratory function and prolonged the lifespan of mice with mitochondrial disease, and clinical trials are ongoing in patients with mitochondrial disease [20,106].

Moreover, plants and their extracts, along with vitamins, have shown promising therapeutic potential against DKD due to their ability to modulate oxidation and inflammation, which are key factors in the development and progression of DKD. For example, essential oils derived from herbs such as *Embelia ribes* Burm have been reported to exhibit antioxidant properties [107,108]. Vitamins C and E, as well as allicin from garlic (*Allium sativum* L.) and proanthocyanidin extract from grape (*Vitis vinifera*) seeds, also have antioxidant, anti-inflammatory and/or anti-diabetic effects [109,110,111]. These candidates are in the preclinical research stage; therefore, there are high expectations for further research and development.

## 5. Effects of H_2_ on Various Renal Diseases and Vascular Endothelial Function

Herein, we investigated the literature for the preventive and therapeutic effects of H_2_ in animal renal disease models and human renal diseases [21,22,23,24,25,26,27,28,29,30,31,32,33,34,35,36,37,38,39,40,41,42,43,44,45,46,47]. The following is a summary of studies that reported the effects of H_2_ on I/R injury, transplantation, CKD, drug-induced kidney injury, kidney stones, renal fibrosis, sepsis-related acute kidney injury (AKI), peritoneal dialysis (PD), and hemodialysis (HD) (Table 1).

Normal vascular endothelial cells can dilate and constrict blood vessels, proliferate and anti-proliferate vascular smooth muscle cells, coagulate and anti-coagulate blood, inflame and anti-inflame, and oxidize and anti-oxidize [112]. The balance of these opposing effects maintains vascular tone and regulates and maintains vascular structure [112]. However, oxidative stress from ROS and RNS induces vascular endothelial injury, and this injury is a factor in the development of atherosclerosis. Furthermore, this atherosclerosis is a risk factor for inducing CKD, including DKD, and cardiovascular diseases [1,2,4]. Therefore, this chapter also provides an overview of the literature on the effects of H_2_ on vascular endothelial function.

### 5.1. Effects on Renal Disease Models in Animals

#### 5.1.1. Ischemia-Reperfusion Injury

Renal I/R injury is an important cause of AKI, a factor in the development of CKD. Shingu et al. investigated the protective effects of H_2_-rich saline solution (HRS) on renal I/R injury in rats. HRS improved mitochondrial morphology and significantly reduced blood urea nitrogen (BUN), creatinine (Cr), and 8-hydroxydeoxyguanosine (8-OHdG) [21].

Similarly, in a rat renal I/R injury model, Wang et al. found that HRS significantly suppressed BUN, Cr, malondialdehyde (MDA), 8-OHdG, tumor necrosis factor-α (TNF-α), interleukin (IL)-1β, IL-6, and myeloperoxidase (MPO), and significantly increased tissue SOD and CAT activities [22].

Li et al. also examined the effects of HRS on a rat model of renal I/R injury and showed that HRS significantly reduced renal tissue stromal congestion, edema, and hemorrhage, as well as BUN, Cr, B-cell/CLL lymphoma 2 (Bcl-2), caspase-3, -8, and -9, IL-6, and TNF-α, while Bcl-2-associated x (Bax) was significantly increased [23]. Furthermore, they reported that the protective effects of HRS may be attributed to its anti-apoptotic and anti-inflammatory effects.

Chen et al. investigated the protective effects of HRS in an I/R-induced AKI mouse model and showed that it significantly reduced renal tissue fibrosis, BUN, and Cr and increased the Klotho levels of anti-aging genes [24]. Moreover, they demonstrated that HRS increased damage-regulated autophagy modulator (Beclin-1) and microtubule-associated protein light chain 3-II (LC3-II) [24]. They suggested that HRS exerted its protective effects through the maintenance of Klotho expression and activation of autophagy in the kidney.

Xu et al. investigated the effects of HRS on a rat model of renal I/R injury and reported that HRS significantly decreased BUN, Cr, MDA, and 8-OHdG and increased HO-1 gene expression and SOD activity [25]. They suggested that HRS ameliorated renal I/R injury in rats by reducing oxidative stress and increasing HO-1 gene expression.

#### 5.1.2. Transplantation

ROS are involved in the development of interstitial fibrosis and tubular atrophy in chronic allograft nephropathy (CAN). Cardinal et al. investigated the effects of drinking H_2_-rich water (HRW) in a rat allogeneic renal transplantation model and found that HRW improved graft function by decreasing BUN, Cr, and urinary protein, slowed CAN progression, decreased MDA, TNF-α, and IL-6, and further prolonged overall survival [26]. HRW also decreased the activation of inflammatory signaling pathways, such as mitogen-activated protein kinase (MAPK), suggesting its effectiveness at preventing CAN and prolonging renal allograft survival [26].

I/R injury is unavoidable in renal transplantation and affects both short- and long-term allograft survival rates. Abe et al. examined the inhibitory effects of H_2_-rich University of Wisconsin (HRUW) solution on I/R injury in a rat renal allograft model and demonstrated that it reduced MDA and 8-OHdG in renal allografts and decreased the numbers of tubular terminal transferase dUTP nick-end labeling (TUNEL)-stained cells and ED-1-positive cells in renal tubules [27]. It also reduced Cr and urinary protein, thereby improving renal function and prolonging recipient survival, which suggested that HRUW alleviated tubular damage and, in turn, reduced the development of interstitial fibrosis [27].

On the other hand, AKI has a significant impact on the survival of liver transplant recipients. Du et al. investigated the protective effects of HRS on AKI after orthotropic liver transplantation in rats and showed that HRW reduced histological damage and decreased BUN, Cr, MDA, and SOD [28]. At the same time, HRS significantly ameliorated apoptosis by suppressing caspase-3 and cytochrome c expression. Furthermore, the expression of Beclin-1 and LC3-II was up-regulated [28]. Chloroquine, an autophagy inhibitor, counteracted the protective effects of HRS [28]. These findings suggested that HRS prevents AKI by reducing apoptosis and activating autophagy.

#### 5.1.3. Chronic Kidney Disease

The Dahl salt-sensitive (SS) rat is a CKD model animal that develops elevated blood pressure and kidney damage with aging. Zhu et al. investigated the effects of H_2_-dissolved electrolyte water (EW) on ischemia-induced cardiorenal injury in the Dahl SS rat [29]. Rats were fed EW or filtered water (FW), after which they underwent unilateral renal I/R. The control group receiving FW showed significant increases in MCP-1, methylglyoxal, and BUN [29]. In a histological examination of the kidneys and heart, significant increases in nitrotyrosine staining were detected in control rats [29]. However, these findings were significantly improved in EW-treated rats, suggesting the potential of EW to prevent CKD [29].

Zhu et al. also examined the effects of EW and FW on age-related cardiorenal injury in Dahl SS rats [30]. Albuminuria and cardiac remodeling increased in the FW group. Histologically, significant age-related changes were observed in the kidney and heart; however, these changes were significantly reduced and MDA and nitrotyrosine decreased in the EW group [30].

Xin et al. investigated the protective effects of HRW on renal damage in spontaneously hypertensive rats (SHRs). HRW significantly reduced BUN and Cr, decreased ROS production, increased SOD, GPX, and CAT activities, and inhibited NADPH oxidase activity in SHR [31]. It also suppressed the expression of TNF-α, IL-6, and IL-1β. Moreover, HRW exerted ameliorative effects on mitochondrial morphology and function, including the suppression of mtROS production and mitochondrial swelling, and increased ATP production [31].

#### 5.1.4. Drug-Induced Renal Injury

Cisplatin is an anticancer drug that is widely used in the treatment of a broad range of tumors; however, its application is limited by oxidative stress-induced nephrotoxicity. Nakashima-Kamimura et al. reported that when mice inhaled H_2_ gas or drank HRW, H_2_ reduced renal injury without impairing the anti-tumor activity of cisplatin [32]. In other words, H_2_ gas or HRW improved cisplatin-induced mortality and weight loss, ameliorated renal histological damage, and restored Cr and BUN [32].

Li et al. investigated the efficacy of HRW in a rat model of iron nitrilotriacetate-induced renal injury [33]. HRW decreased Cr, BUN, MDA, ONOO− production, and NADPH oxidase activity and increased CAT activity. HRW ameliorated mitochondrial dysfunction and oxidative stress, including kidney mitochondrial swelling, decreased ATP production, and increased mtROS production [33]. HRW also suppressed inflammation as indicated by the decreased expression of nuclear factor-κB (NF-κB), IL-6, and monocyte chemotactic protein-1 (MCP-1) in the kidney [33]. Furthermore, HRW suppressed vascular endothelial growth factor (VEGF) expression and signal transducer and activator of transcription 3 (STAT3) phosphorylation, thereby reducing the incidence of renal cell carcinoma and inhibiting tumor growth [33].

Oxidative stress induced by cyclosporin A is a major cause of chronic kidney injury. Lu et al. examined the mitigating effects of HRW on cyclosporine A-induced renal injury in rats and found that it decreased ROS production, MDA, and Keap1 and increased the expression of Nrf-2 and HO-1 [34]. They suggested that the effects of HRW involved the amelioration of oxidative stress through the activation of the Keap1/Nrf-2 signaling pathway.

#### 5.1.5. Renal Stones

Peng et al. evaluated the protective effects of H_2_ gas against glyoxylate-induced renal calcium oxalate (CaOx) crystal deposition in mice and reported that it decreased MDA and 8-OHdG levels and increased SOD, GSH, and CAT activities [35]. They also showed that H_2_ gas reduced MCP-1 and increased IL-10 expression, indicating that H_2_ gas exerted protective effects against renal stone disease by reducing renal crystallization, renal oxidative damage, and inflammation [35].

#### 5.1.6. Renal Fibrosis

Xu et al. examined the efficacy of HRS in a model of renal fibrosis induced by unilateral ureteral obstruction (UUO) in rats and showed that it significantly improved the renal injury score, apoptosis index, stromal fibrosis, and macrophage infiltration in renal tissue [36]. Additionally, HRS reduced MDA levels and increased SOD activity.

Furthermore, Xing et al. investigated the efficacy of HRW in a mouse model of renal fibrosis caused by UUO and showed that it suppressed Cr, BUN, and renal fibrosis [37]. They also examined the inhibitory effects of HRW on renal epithelial–mesenchymal transition (EMT) induced by transforming growth factor-β1 (TGF-β1) using human renal proximal tubular epithelial cells and showed that HRW abolished EMT and restored decreases in the expression of sirtuin-1 (Sirt1) [37]. Sirtinol, the inhibitor of Sirt1, abolished the inhibitory effects of HRW on EMT, indicating that HRW ameliorated renal injury and fibrosis by regulating Sirt1 [37].

Congenital obstructive nephropathy is commonly implicated in the pathophysiology of CKD, and the release of ROS contributes to the exacerbation of renal fibrosis. Mizutani et al. evaluated the efficacy of HRW in a rat model of UUO-induced renal injury [38]. HRW suppressed tubulointerstitial injury and reduced the area of interstitial fibrosis and frequency of TGF-β1-positive cells [38]. In addition, HRW restored decreases in Klotho mRNA expression.

#### 5.1.7. Sepsis-Related Acute Kidney Injury

Liu et al. investigated the combined effects of early infusion resuscitation and H_2_ gas on AKI occurring during septic shock in rats induced by lipopolysaccharide, and showed that the combination of both reduced BUN and Cr [39]. It also decreased MDA and reduced renal TNF-α and IL-6 levels more than infusion resuscitation alone [39]. These findings indicated that early infusion resuscitation combined with H_2_ gas exerted stronger protective effects against AKI.

Yao et al. examined the protective effects of the aerosol inhalation of HRS in a model of sepsis-related AKI in mice induced by cecum ligation and puncture [40]. AKI occurred during the early stage of sepsis, as evidenced by increases in BUN and Cr, renal fibrosis, and renal tubular epithelial cell apoptosis, and was accompanied by macrophage infiltration and the generation of inflammatory cytokines (IL-6 and TNF-α) [40]. In contrast, HRS aerosol inhalation increased the mRNA levels of anti-inflammatory cytokines (IL-4 and IL-13) and enhanced the generation of anti-inflammatory cytokines (IL-10 and TGF-β) in renal tissues, suggesting the utility of HRS aerosol inhalation for renal protection and the attenuation of inflammation in septic AKI [40].

#### 5.1.8. Others

Guo et al. investigated the efficacy of HRS in a rat model of severe burn-induced early AKI and reported that HRW improved renal function (BUN and Cr) and attenuated tubular apoptosis [41]. Furthermore, the mechanisms underlying the AKI-ameliorating effect of HRW involved the inhibition of oxidative stress-induced apoptosis and inflammation, and these effects appeared to be mediated through the regulation of MAPK and nuclear factor (NF)-κB signaling pathways [41].

Shi et al. examined the protective effects of HRS on AKI and the underlying mechanisms in sodium taurocholate-induced acute pancreatitis in rats [42]. The findings obtained showed that HRS prevented the progression of the inflammatory cascade and alleviated oxidative damage in the kidney by inhibiting NF-κB activation and removing ROS [42].

Furthermore, Guan et al. analyzed the protective effects of H_2_ gas on renal damage caused by chronic intermittent hypoxia (CIH) in rats in terms of oxidative stress, autophagy, and ER stress [43]. H_2_ gas also improved renal function in rats with CIH, and alleviated histological damage, oxidative stress, and apoptosis. They also found that H_2_ gas ameliorated CIH-induced renal injury by suppressing oxidative stress-dependent MAPK activation, thereby reducing ER stress, and activating autophagy [43].

### 5.2. Effects on Human Renal Diseases

#### 5.2.1. Peritoneal Dialysis

Oxidative stress derived from glucose degradation products is responsible for peritoneal degradation in patients with PD. Terawaki et al. investigated the effects of a H_2_-enriched dialysate (HED) on peritoneal oxidative stress in six patients with PD [44]. Based on the findings showing that the percentage of reduced albumin was higher and the percentage of oxidized albumin was lower in the effluent and serum of PD patients treated with HED than with the standard dialysate, HED appeared to reduce peritoneal and systemic oxidative stress [44].

#### 5.2.2. Hemodialysis

Nakayama et al. developed a dialysis system using a dialysate dissolved in H_2_ gas and investigated the efficacy of HED in 21 patients with HD. HED significantly reduced systolic blood pressure before and after dialysis [45]. Moreover, it significantly decreased MCP-1 and MPO, suggesting its potential to control uremia by attenuating inflammation [45].

Terawaki et al. investigated the effects of HED on oxidative stress in eight HD patients in a crossover study using a standard dialysate (SD) and HED and showed that HED significantly reduced the mean percentage of oxidized albumin in serum at the exit of the dialysis system more than SD [46].

Sokawa et al. examined the effects of H_2_ gas on oxidative stress and inflammatory responses in six HD patients [47]. The inhalation of H_2_ gas three times a week for two weeks did not affect the biological antioxidant potential (BAP). However, it significantly reduced diacron-reactive oxygen metabolites (d-ROMs) and C-reactive protein (CRP), and these effects persisted for two weeks after the discontinuation of H_2_ gas inhalation, indicating that the inhalation of H_2_ gas attenuated oxidative stress and inflammatory responses in HD patients [47].

### 5.3. Effects on Vascular Endothelial Function

Jiang et al. induced vascular endothelial cell injury in cultured rat blood vessels by adding advanced glycation end products (AGEs) and examined the protective effect of hydrogen-rich medium (HRM) [113]. They showed that HRM significantly decreased ROS, increased antioxidant enzymes, and decreased apoptosis [113]. These results indicate that H_2_ inhibits vascular endothelial injury induced by AGE through its inhibitory effects on oxidative stress and apoptosis.

Ohsawa et al. investigated the inhibitory effect of HRW on atherosclerosis by drinking HRW to apolipoprotein E (ApoE)-deficient mice [114]. They showed that the lesion areas of atherosclerosis were significantly reduced in mice in the HRW group [114]. In addition, an inhibition of macrophage accumulation and reduction in oxidative stress were observed in the arterial tissues of mice in the HRW group, suggesting that drinking HRW may prevent atherosclerosis in ApoE-deficient mice [114].

Truong et al. examined the effects of H_2_ gas on vascular endothelial glycocalyx in a heat stroke-induced rat model [115]. They showed that H_2_ gas improved the survival rate of heat stroke-induced rats and inhibited the detachment of vascular endothelial glycans. Furthermore, H_2_ gas decreased MDA and TNF-α levels and increased SOD levels [115]. These results indicate that H_2_ gas attenuates vascular endothelial glycocalyx injury through antioxidant and anti-inflammatory effects.

Song et al. investigated the effect of HRW on improving lipid metabolism in 20 patients with metabolic syndrome [116]. They conducted a comparative experiment using high-density lipoprotein (HDL) from serum collected from patients before and after drinking HRW. They demonstrated that HDL after drinking HRW inhibited low-density lipoprotein (LDL) oxidation, inhibited monocyte adhesion to vascular endothelial cells, promoted cholesterol extraction from macrophages that had taken up oxidized LDL, and inhibited the apoptosis of vascular endothelial cells [116]. These results indicate that HRW improves lipid metabolism by improving HDL function in vascular endothelial cells.

Sakai et al. investigated the effects of HRW on vascular endothelial function in healthy subjects [117]. Vascular endothelial function was assessed by flow-mediated dilation (FMD) measurements of arteries. Comparing FMD before and after the drinking of placebo water or HRW alone, FMD decreased in 18 placebo group subjects, while FMD increased in 16 HRW subjects, and this change was a significant improvement in vascular endothelial function [117]. These results indicate that H_2_ may maintain the NO-mediated vasomotor response.

Ishibashi et al. also examined the effects of 2 weeks of drinking HRW on vascular endothelial function in healthy subjects in a randomized controlled trial [118]. Vascular endothelial function was assessed by measuring the reactive hyperemia index (RHI) of finger vessels using peripheral arterial tonometry. They showed that the HRW group (34 subjects) showed a significant improvement in RHI compared to the placebo group (34 subjects), with the effect being particularly pronounced 24 h after the first intake of HRW and after daily intake for 2 weeks [118]. These results suggest that H_2_ improves endothelial function in arteries or arterioles.

In conclusion, this literature review suggests that H_2_ may potentially safeguard against endothelial dysfunction and hinder the progression of DKD to cardiovascular disease by mitigating oxidative stress, inflammation, and apoptosis.

## 6. Mechanism of Action of H_2_ on Renal Disease

**Figure 4:**
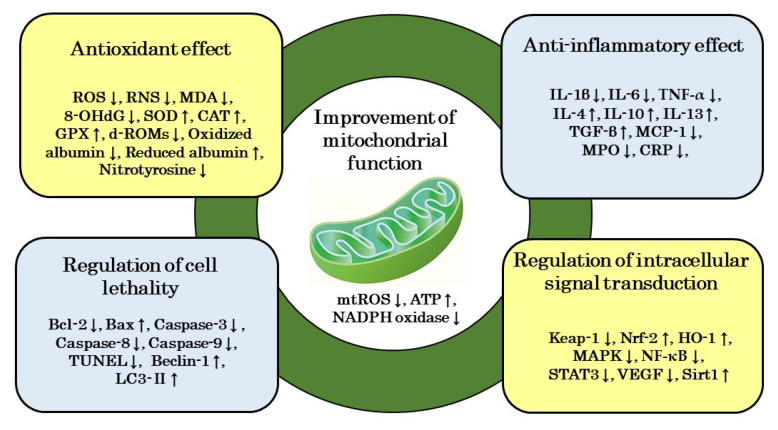
Possible mechanisms of action of H_2_ in renal disease. Its effects are mainly categorized as an improvement in mitochondrial function, antioxidant and anti-inflammatory effects, and the regulation of cell lethality and intracellular signal transduction. Bax: Bcl-2-associated x; Beclin-1: damage-regulated autophagy modulator; Bcl-2: B-cell/CLL lymphoma 2; CAT: catalase; CRP: C-reactive protein; d-ROMs: diacron-reactive oxygen metabolites; GPX: glutathione peroxidase; H_2_: molecular hydrogen; HO-1: heme oxygenase-1; 8-OHdG: 8-hydroxydeoxyguanosine; IL: interleukin; Keap1: Kelch-like ECH-associated protein 1; LC3- II: microtubule-associated protein light chain 3-II; MPO: myeloperoxidase; MAPK: mitogen-activated protein kinase; MCP-1: monocyte chemotactic protein-1; MDA: malondialdehyde; NADPH: nicotinamide adenine dinucleotide phosphate; NF-κB: nuclear factor-κB; Nrf-2: nuclear factor erythroid-related factor 2; ROS: reactive oxygen species; SOD: superoxide dismutase; STAT3: signal transducer and activator of transcription 3; Sirt1: sirtuin-1; TNF-*α*: tumor necrosis factor-α; TUNEL: tubular terminal transferase dUTP nick-end labeling; TGF-β: transforming growth factor-β1; VEGF: vascular endothelial growth factor; ↑: increase; ↓: decrease.

The preventive and therapeutic effects of H_2_ on renal diseases have been reported in many studies in which H_2_ improved histological injuries and reduced serum BUN and Cr and urinary protein. On the other hand, based on a literature review, the mechanism of action of H_2_ on renal diseases may be mainly categorized as an improvement in mitochondrial function, antioxidant and anti-inflammatory effects, and the regulation of cell lethality and intracellular signal transduction. These mechanisms are not independent of each other; they interact to form the complex mechanism of H_2_ (Figure 4).

### 6.1. Improvement in Mitochondrial Function

Transmission electron microscopy is a commonly used method to examine morphological changes in mitochondria. The effects of H_2_ on mitochondrial morphology showed that H_2_ ameliorated mitochondrial swelling [21,31]. It also inhibited mtROS production, enhanced ATP production, and decreased NADPH oxidase activity [31,33], suggesting that its mechanisms of action involve ameliorative effects on mitochondrial morphology and function.

### 6.2. Antioxidant Effects

Using fluorescent reagents, H_2_ was shown to reduce the fluorescence intensity of ROS or RNS in renal tissue [33,34]. H_2_ also decreased MDA, a marker of lipid oxidation, and 8-OHdG, a marker of DNA oxidation [21,22,25,26,27,28,30,33,34,35,36,39]. The activities of antioxidant enzymes in renal tissue, such as SOD, CAT, and GPX, are used as markers to assess antioxidant activity; H_2_ increased the activities of these antioxidant enzymes [22,25,28,31,33,35,36]. Furthermore, oxidized albumin and d-ROMs are used as oxidative markers in human clinical studies and reduced albumin and BAP as antioxidant markers; H_2_ decreased oxidized albumin and d-ROMs and increased reduced albumin in PD or HD patients [44,46,47]. On the other hand, ONOO− modifies tyrosine residues exposed on the protein surface to produce nitrotyrosine. This nitrotyrosine has attracted attention as a nitrosative stress marker in various inflammatory diseases; H_2_ reduced ONOO− and inhibited nitrotyrosine production, thereby ameliorating oxidative stress, nitrosative stress, and inflammation [29,30]. Therefore, experimental findings indicate that H_2_ ameliorates renal injury through its antioxidant properties.

### 6.3. Anti-Inflammatory Effects

IL-1β, IL-6, and TNF-α are inflammatory cytokines, while IL-4, IL-10, IL-13, and TGF-β are anti-inflammatory cytokines. In experiments to examine the mRNA expression or protein levels of these cytokines in animal models, H_2_ decreased the former while simultaneously increasing the latter [22,23,26,31,33,35,39,40]. In addition, MCP-1 and MPO have been used as markers of macrophage infiltration and inflammatory responses. In animal models of renal disease and HD patients, H_2_ decreased MCP-1 and MPO levels [33,35,45] and reduced CRP in human HD patients [47]. These findings suggest that H_2_ exerts protective effects against renal injury through its anti-inflammatory properties.

### 6.4. Regulation of Cell Lethality

Bcl-2 is a protein that promotes apoptosis, while Bax is a protein that inhibits apoptosis. The protease family, which is involved in apoptosis, includes caspase-3, -8, and -9. In addition, the TUNEL staining method is used to examine DNA fragmentation due to apoptosis. In a renal disease model, H_2_ not only suppressed Bcl-2 gene expression and increased Bax gene expression, but also suppressed the expression of caspase-3, -8, and -9 [23,28]. H_2_ also reduced TUNEL-positive cells in renal tubules [27]. On the other hand, Beclin-1 and LC3-II have been identified as regulators and markers of autophagy; H_2_ not only exerted ameliorative effects on renal injury, but also increased the expression of Beclin-1 and LC3-II [24,28]. However, chloroquine, an autophagy inhibitor, nullified the effects of H_2_ [28]. These findings indicate that H_2_ regulates cell lethality by inhibiting apoptosis and activating autophagy.

### 6.5. Regulatory Effects of Signal Transduction

Previous findings demonstrated that the mechanisms underlying the protective effects of H_2_ on renal diseases involved a decrease in Keap1 levels and an increase in Nrf-2 and HO-1 gene expression [25,34]. Therefore, the attenuation of oxidative stress and enhanced biological defense functions through the activation of the Keap1/Nrf-2 signaling pathway are involved in the effects of H_2_. Moreover, the suppression of signaling pathways, such as MAPK and NF-κB, were shown to play a role in the antioxidative and anti-inflammatory effects of H_2_ [26,33,41,43]. Furthermore, the inhibition of signaling pathways for STAT3 phosphorylation and VEGF expression as well as the activation of that for Sirt1 expression contributed to the effects of H_2_ [33,37,41]. Since sirtinol, a Sirt1 inhibitor, nullified the effects of H_2_, Sirt1 may be involved in the protective effects of H_2_ on renal disease [37]. Therefore, H_2_ exerts its ameliorative effects on renal injury through the activation or suppression of various signaling pathways.

## 7. Therapeutic Potential of H_2_ for Diabetic Kidney Disease

### 7.1. Therapeutic Potential of H_2_ in the Etiology of Diabetic Kidney Disease

**Figure 5:**
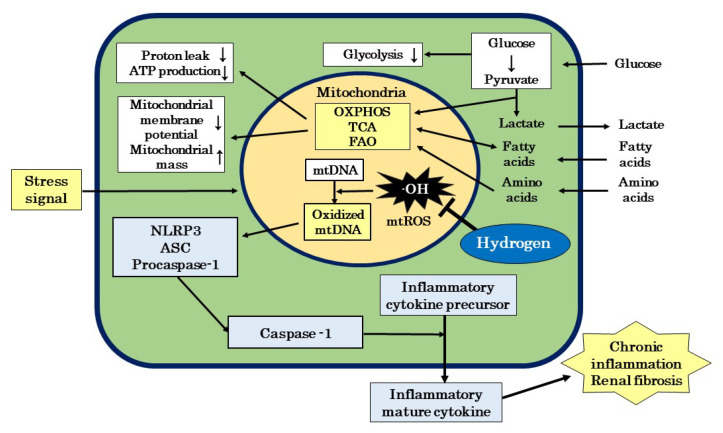
A possible mechanism by which H_2_ ameliorates mitochondrial dysfunction in patients with DKD. H_2_ ameliorates mitochondrial dysfunction by scavenging ·OH and blocks the cascade from NLRP3 activation to the release of inflammatory cytokines, which attenuates chronic inflammation and fibrosis in the kidney. ASC: apoptosis-associated speck-like protein containing a caspase recruitment domain; ATP: adenosine triphosphate; DKD: diabetic kidney disease; FAO: fatty acid oxidation; mtDNA: mitochondrial DNA; mtROS: mitochondrial reactive oxygen species; NLRP3: nucleotide-binding and oligomerization domain-like receptor family pyrin domain-containing 3; ∙OH: hydroxyl radicals; OXPHOS: oxidative phosphorylation; TCA: tricarboxylic acid cycle.

Inflammation is both a cause and consequence of the onset and progression of DKD. Inflammation is triggered by inflammatory cytokines released by innate immunity. Pathogens such as viruses and bacteria, substances produced when the body is damaged, and irritants in the environment serve as inflammation-inducing signals [119,120]. These external signals cause mitochondrial dysfunction and induce the excessive production of ROS [121,122,123]. Excessive mtROS production in mitochondria results in the release of oxidized mtDNA into the cytoplasm, which, in turn, leads to the formation of the nucleotide-binding and oligomerization domain-like receptor family pyrin domain-containing 3 (NLRP3) inflammasome [121,122,123]. The NLRP3 inflammasome then activates caspase-1, which induces the release of mature inflammatory cytokines from immune cells, such as macrophages and neutrophils, resulting in inflammation (Figure 5) [121,122,123].

On the other hand, other intracellular oxidative stress responses are also involved in the induction of inflammation. The stress-activated protein kinase (SAPK) pathway plays a central role in this oxidative stress response [124]. Activation of the SAPK pathway by oxidative stress stimuli induces the expression of various genes involved in the stress response, which ultimately induces inflammation and cell death [124]. Furthermore, ROS are strongly involved in vascular inflammation. Excess ROS activate redox transcription factors such as NF-κB and activator protein 1, resulting in monocyte invasion of the vessel wall and increased inflammatory cytokine production [125,126]. However, the presence of angiotensin II, oxidized LDL, and inflammatory cytokines activates NADPH oxidase, which induces inflammation under conditions of oxidative stress due to excess ROS [125,126]. Thus, oxidative stress, inflammation, and vascular endothelial dysfunction may be interrelated.

Advanced proteinuria is a progressive risk factor for the formation of interstitial lesions in DKD. In tubulointerstitial lesions associated with advanced proteinuria, tubular damage is induced by the excessive reabsorption of free fatty acids, a mechanism that involves the activation of the NLRP3 inflammasome through mitochondrial damage [127]. In addition, mineralocorticoid receptor (MR) activation is closely involved in renal inflammation and fibrosis, and MR activation has been shown to induce the production of mtROS [128]. Furthermore, the activation of caspase-1 in glomerular epithelial cells may be important for the formation of glomerulosclerotic lesions in DKD [129]. On the other hand, many studies that investigated the efficacy of H_2_ in inflammatory disease models suggested that the inhibition of mtROS production by H_2_ is involved in the mechanism by which H_2_ suppresses acute and chronic inflammation [130,131,132,133,134,135]. Therefore, we proposed a possible mechanism for the efficacy of H_2_ against inflammatory disease models that involves H_2_ reducing ∙OH and suppressing oxidative damage to mtDNA, which, in turn, inhibits a series of signaling pathways from activation of the NLRP3 inflammasome to the release of inflammatory cytokines [48]. H_2_ may ameliorate the formation of stromal lesions in patients with DKD by suppressing NLRP3 inflammasome activation and ameliorating chronic inflammation and fibrosis in the kidney (Figure 5).

Diabetic peripheral neuropathy (DNP) is another serious diabetic complication similar to DKD. Jiao et al. investigated the efficacy of HRS against DNP in a streptozotocin-induced diabetic rat model and showed that it significantly suppressed the behavioral, biochemical, and molecular biological effects of DNP in rats [136]. They also reported that 5-hydroxydecanoate, a selective inhibitor of the mitochondrial ATP-sensitive K^+^ (mitoK_ATP_) channel, partially attenuated the therapeutic effects of HRS [136]. These findings indicate that the mechanism underlying the efficacy of HRS against DNP involves a protective effect on mitochondria through the activation of the mitoK_ATP_ pathway. Furthermore, we reported that the mechanism by which H_2_ is effective in animal models of various diseases, and human chronic inflammatory diseases, such as the “sequelae” of coronavirus infection 2019 (COVID-19) called post-COVID-19 and myalgic encephalomyelitis/chronic fatigue syndrome (ME/CFS), may involve an improvement in mitochondrial function [49]. These findings suggest that the therapeutic effects of H_2_ in patients with DKD involve an improvement in mitochondrial function (Figure 5).

### 7.2. Prospects for H_2_ as a Therapeutic Substance for Diabetic Kidney Disease

The therapeutic effects of H_2_ have been observed in a wide range of diseases, and its efficacy has been reported in more than 130 clinical papers. Since no side effects of H_2_ were observed in these studies, H_2_ is a medical gas with excellent efficacy and safety [10,54,137]. In addition, H_2_ is a convenient gaseous molecule that may be inhaled directly as a gas or dissolved in water or saline solution for drinking or intravenous administration [10,58,137]. H_2_ also has excellent pharmacokinetic and intracellular kinetic characteristics [10,58,138]. Mitochondrial dysfunction, oxidative stress, inflammation, and cell lethality are closely related to the onset and progression of DKD; therefore, H_2_ may be effective against DKD. The mechanisms by which H_2_ exhibits efficacy in various animal renal disease models and human dialysis patients, as well as our previous findings, provide evidence for the therapeutic potential of H_2_ for DKD [21,22,23,24,25,26,27,28,29,30,31,32,33,34,35,36,37,38,39,40,41,42,43,44,45,46,47,48,49]. Furthermore, a literature review examining the effects of H_2_ on vascular endothelial function showed that H_2_ may inhibit the progression of DKD to cardiovascular disease by suppressing oxidative stress, inflammation, and apoptosis [113,114,115,116,117,118]. Large-scale clinical trials are needed to demonstrate this potential.

On the other hand, several limitations have been recognized in the study of medical applications of H_2_. A recent study reported that oxidized porphyrins function as target molecules of H_2_ and catalyze the reaction of H_2_ with ·OH [11]. However, the target molecules of H_2_ are still in the early stages, having only been partially elucidated [11]. Furthermore, information on dosages and usages for individual diseases, including optimal H_2_ concentrations, daily dosages, and durations of intake, remains unclear. Moreover, while the improvement in mitochondrial function by H_2_ may exert therapeutic effects on DKD, other mechanisms of H_2_ may be involved. In addition, the majority of DKD models used in animal studies exhibit minor clinical symptoms, which diverge from the clinical symptoms of human DKD. Therefore, further studies on the optimal dosage and usage of H_2_ for individual diseases, the mechanisms of action of H_2_, including its target molecules, and the development of animal models of DKD are warranted.

## 8. Conclusions

H_2_ has demonstrated efficacy in various animal models of renal disease and in dialysis patients, and the mechanisms of action of H_2_ include mitochondrial improvement, antioxidant and anti-inflammatory effects, and the regulation of cell lethality and intracellular signaling. Mitochondrial dysfunction, oxidative stress, inflammation, cell lethality, and intracellular signaling are involved in the pathogenesis and progression of DKD. Our analysis of the literature reporting the efficacy of H_2_ in animal models of renal disease and in human dialysis patients in this article suggests that H_2_ may have therapeutic potential in patients with DKD. This therapeutic potential of H_2_ is supported by our mechanistic analysis in this article including the efficacy of H_2_ in human chronic inflammatory diseases, such as post-COVID-19 and ME/CFS. Therefore, this review will provide an opportunity to consider the possibility of clinical trials of H_2_ against DKD. Future large-scale clinical trials are needed to confirm the effects of H_2_ on DKD.

## Figures and Tables

**Table 1:** Summary of effects of molecular hydrogen (H_2_) in animal renal disease models and human renal diseases.

Species	Type of H_2_	Effects of H_2_		Ref.
		Diseases	Changes in Biomarkers	
Rats	HRS	AKI	Swelling of Mt.↓, BUN↓, Cr↓, 8-OHdG↓	[21]
Rats	HRS	I/R injury	BUN↓, Cr↓, MDA↓, 8-OHdG↓, TNF-*α*↓, IL-1β↓, IL-6↓, MPO↓, SOD↑, CAT↑	[22]
Rats	HRS	I/R injury	Tissue injury↓, BUN↓, Cr↓, Bcl-2↓, Caspase-3, -8, and -9↓, IL-6↓, TNF-*α*↓, Bax↑	[23]
Mice	HRS	AKI	Tissue injury↓, BUN↓, Cr↓, Klotho↑, Beclin-1↑, LC3- II↑	[24]
Rats	HRS	I/R injury	BUN↓, Cr↓, MDA↓, 8-OHdG↓, HO-1↑, SOD↑	[25]
Rats	HRW	Renal Transplantation	Overall survival↑, BUN↓, Cr↓, Urinary protein↓, MDA↓, TNF-α↓, IL-6↓, MAPK↓	[26]
Rats	HRUW	Renal Transplantation	Overall survival↑, MDA↓, 8-OHdG↓, TUNEL-stained cells↓, ED-1-positive cells↓, Cr↓, Urinary protein↓	[27]
Rats	HRW	AKI	BUN↓, Cr↓, MDA↓, SOD↑, Caspase-3↓, Cytochrome C↓, Beclin-1↑, LC3- II↑	[28]
Rats	EW	CKD	MCP-1↓, Methylglyoxal↓, BUN↓, Nitrotyrosine staining↓	[29]
Rats	EW	CKD	Age-related histological changes↓, albuminuria↓, cardiac remodeling↓, MDA↓, nitrotyrosine staining↓	[30]
Rats	HRW	CKD	BUN↓, Cr↓, ROS↓, SOD↑, GPX↑, CAT↑, NADPH oxidase↓, TNF-α↓, IL-6↓, IL-1β↓	[31]
Mice	HRW/H_2_ gas	Cisplatin-induced injury	Histological injury ↓, BUN↓, Cr↓	[32]
Rats	HRW	Fe-NTA-induced injury	Cr↓, BUN↓, MDA↓, ONOO−↓, NADPH oxidase↓, CAT↑, mtROS↓, NF-κB↓, IL-6↓, MCP-1↓, VEGF↓, STAT3↓	[33]
Rats	HRW	Cyclosporin A-induced injury	ROS↓, MDA↓, Keap1↓, Nrf-2↑, HO-1↑	[34]
Mice	H_2_ gas	Renal stones	MDA↓, 8-OHdG↓, SOD↑, GSH↑, CAT↑, MCP-1↓, IL-10↑	[35]
Rats	HRS	Renal fibrosis	Injury score↓, apoptosis index↓, stromal fibrosis↓, MDA↓, SOD↑	[36]
Mice	HRW	Renal fibrosis	Cr↓, BUN↓, fibrosis↓, EMT↓, Sirt1↑	[37]
Rats	HRW	Renal fibrosis	Fibrosis↓, TGF-β1-positive cells↓, Klotho↑	[38]
Rats	H_2_ gas	Sepsis-related AKI	BUN↓, Cr↓, MDA↓, TNF-α↓, IL-6↓	[39]
Mice	HRS	Sepsis-related AKI	IL-4 ↑, IL-13↑, IL-10↑, TGF-β↑	[40]
Rats	HRS	Burn-induced AKI	BUN↓, Cr↓, tubular apoptosis↓, inflammation↓, MAPK↓, NF-κB↓	[41]
Rats	HRS	AKI	NF-κB↓, ROS↓	[42]
Rats	H_2_ gas	Hypoxia-induced injury	Renal function↑, histological damage↓, oxidative stress↓, apoptosis↓, MAPK↓	[43]
Humans	HED	PD	Reduced albumin↑, oxidized albumin↓	[44]
Humans	HED	HD	SBP↓, MCP-1↓, MPO↓	[45]
Humans	HED	HD	Oxidized albumin↓	[46]
Humans	H_2_ gas	HD	d-ROMs↓, CRP↓	[47]

AKI: acute kidney disease; Bax: Bcl-2-associated x; Beclin-1: damage-regulated autophagy modulator; Bcl-2: B-cell/CLL lymphoma 2; BUN: blood urea nitrogen; CAT: catalase; Cr: creatinine; CRP: C-reactive protein; CKD: chronic kidney disease; ↓: decrease; d-ROMs: diacron-reactive oxygen metabolites; EMT: epithelial–mesenchymal transition; GPX: glutathione peroxidase; H_2_: molecular hydrogen; HRS: hydrogen-rich saline; HRW: hydrogen-rich water; HRUW: H_2_-rich University of Wisconsin; HED: H_2_-enriched dialysate; HD: hemodialysis; HO-1: heme oxygenase-1; 8-OHdG: 8-hydroxydeoxyguanosine; Fe-NTA: iron nitrilotriacetate; ↑: increase; IL: interleukin; Keap1: Kelch-like ECH-associated protein 1; LC3-II: microtubule-associated protein light chain 3-II; Mt.: mitochondria; MPO: myeloperoxidase; mtROS: mitochondrial ROS; MAPK: mitogen-activated protein kinase; MCP-1: monocyte chemotactic protein-1; MDA: malondialdehyde; NADPH: nicotinamide adenine dinucleotide phosphate; NF-κB: nuclear factor-κB; Nrf-2: nuclear factor erythroid-related factor 2; PD: peritoneal dialysis; ONOO−: peroxynitrite; Ref.: reference; ROS: reactive oxygen species; SOD: superoxide dismutase; STAT3: signal transducer and activator of transcription 3; Sirt1: sirtuin-1; SBP: systolic blood pressure; TNF-α: tumor necrosis factor-α; TUNEL: tubular terminal transferase dUTP nick-end labeling; TGF-β: transforming growth factor-β1; VEGF: vascular endothelial growth factor.

## Data Availability

No research data were collected.
